# The Physical Factors Influencing Infection

**Published:** 1919

**Authors:** 


***The Physical Factors Influencing Infection.*** By Walton Martin. *Annals of Surgery*, October, 1918.

The gravity of infection is regulated by a number of mechanical conditions which are realized anatomically in certain wounds and not in others. These conditions are:

(1) Pressure at the focus of infection, or point of initial lodgment.

(2) Foreign bodies.

(3) Devitalized and necrotic tissues.

(4) Dead spaces.

Local pressure has been proved, both experimentally and practically, to be an important factor in the initial lodgment and in the persistence of pathogenic bacteria in the tissues. The author refers here to Friedrich's work on the infection in open wounds, the experiments of Schimmelbusch showing the rapidity with which organisms are forced into the wound surface by friction, finally those of Giani, proving that a trifling mechanical violence is sufficient to break the barrier of leukocytes which is formed along the wound surface.

Foreign bodies introduced into the tissues act both as mechanical and chemical irritants, but their main significance is as vehicles or conveyers of infection. In gunshot injuries, particles of earth, etc., are forced into the tissues, each with its modicum of infection, and become anchored there, the flow of lymph and blood being incapable of carrying them out. Policard and Phelip's report on the histological and bacteriological findings about infected bodies is given in detail, together with a chart illustrating the fall in the number of bacteria after the foreign body has been removed.

In every injury there is a period of paralysis of all cellular activity following the shock, and varying in a direct proportion to the degree of the initial violence; there is also cellular death. The amount of necrosis has again a direct relation to mechanical violence, as well as an important relation to the initial lodgment of bacteria and to their persistence in the tissue. Certain organisms live and multiply only provided there is a certain amount of necrotic tissue; others are only pathogenic when this medium is present. In every wound, the necrotic tissue must be eliminated before healing can occur. This elimination is brought about to a very limited extent by autolysis.

Dead spaces furnish favorable conditions for the growth and persistence of bacteria in the tissues.

The author insists upon the importance of the above mentioned conditions and gives the principles forming the basis of the treatment of infected wounds :

(1) Relief of tissue tension and pressure from without.

(2) Mechanical elimination of necrotic and devitalized tissue.

(3) Removal of foreign bodies.

(4) Avoidance of stagnant fluids in dead spaces and obliteration of uncollapsible cavities.

